# A Small Molecule Inhibitor of Signal Peptide Peptidase Inhibits *Plasmodium* Development in the Liver and Decreases Malaria Severity

**DOI:** 10.1371/journal.pone.0005078

**Published:** 2009-04-01

**Authors:** Iana Parvanova, Sabrina Epiphanio, Abdul Fauq, Todd E. Golde, Miguel Prudêncio, Maria M. Mota

**Affiliations:** 1 Unidade de Malária, Instituto de Medicina Molecular, Faculdade de Medicina da Universidade de Lisboa, Lisboa, Portugal; 2 Department of Neuroscience, Mayo Clinic, College of Medicine, Jacksonville, Florida, United States of America; 3 Instituto Gulbenkian de Ciência, Oeiras, Portugal; INSERM U567, Institut Cochin, France

## Abstract

The liver stage of *Plasmodium*'s life cycle is the first, obligatory step in malaria infection. Decreasing the hepatic burden of *Plasmodium* infection decreases the severity of disease and constitutes a promising strategy for malaria prophylaxis. The efficacy of the gamma-secretase and signal peptide peptidase inhibitor LY411,575 in targeting *Plasmodium* liver stages was evaluated both in human hepatoma cell lines and in mouse primary hepatocytes. LY411,575 was found to prevent *Plasmodium*'s normal development in the liver, with an IC_50_ of approximately 80 nM, without affecting hepatocyte invasion by the parasite. *In vivo* results with a rodent model of malaria showed that LY411,575 decreases the parasite load in the liver and increases by 55% the resistance of mice to cerebral malaria, one of the most severe malaria-associated syndromes. Our data show that LY411,575 does not exert its effect via the Notch signaling pathway suggesting that it may interfere with *Plasmodium* development through an inhibition of the parasite's signal peptide peptidase. We therefore propose that selective signal peptide peptidase inhibitors could be potentially used for preventive treatment of malaria in humans.

## Introduction

Malaria is a devastating parasitic disease accounting for 1 to 2 million deaths per year, mostly among children in Sub-Saharan Africa, Asia, Central and South America. During the last two decades, the incidence of malaria has been increasing, largely due to an emergence of parasite variants resistant to the two most widely used drugs, chloroquine and sulphadoxine/pyrimethamine. This fact, taken together with the largely unsuccessful attempts for antimalarial vaccination, makes the development of new drugs against this disease critically important [1].

Malaria is caused by protozoan parasites from the *Plasmodium* genus. *Plasmodium* sporozoites are transmitted to the mammalian host by a mosquito bite and transported with the blood stream to the liver. Once in the liver, the parasites cross the sinusoidal wall, presumably through Kupffer cells, and migrate through several hepatocytes before infecting a final cell, which they enter with formation of a parasitophorous vacuole [2]–[4]. Within the vacuole, the sporozoites develop and produce thousands of merozoites, which are released into the bloodstream and infect erythrocytes [5], [6]. The liver stage of the disease is clinically silent while all pathological manifestations develop during the blood stage [7], [8]. All currently used antimalarial agents, with the exception of primaquine, target blood stage parasites. Drugs against liver stage malaria would block the development of the parasites and prevent pathology. It is therefore crucial to develop novel agents against this stage of infection.

One of the emerging strategies for treatment of malaria is the use of enzymatic inhibitors. A number of enzymes essential for parasite metabolism have been recognized as attractive targets for novel drug development. Inhibitors of the plasmepsin family of aspartyl proteases are already established as potential agents against blood stage malaria through extensive data generated in cell culture and mouse models [9]–[11]. Inhibitors targeting the falcipains, a family of *P. falciparum* cysteine proteases involved in hemoglobin degradation and erythrocyte invasion, have demonstrated potent antimalarial effects and their testing and optimization as antimalarials is under way (reviewed in [12]). Additionally, HIV protease inhibitors already in clinical use were also shown to inhibit growth of *P. falciparum* in culture *P. berghei* in mice [13], [14].

Here we show that the gamma-secretase and signal peptide peptidase (SPP) inhibitor LY411,575, but not the selective gamma-secretase inhibitor (GSI) DAPT, impairs development of *P. berghei in vitro* in hepatoma cells as well as *in vivo* in mouse liver. These data indicate that *Plasmodium* SPP is a potential therapeutic target for malaria, and provide rationale for development of selective *Plasmodium* SPP inhibitors, perhaps based on the LY411,575 scaffold as novel treatments for malaria.

## Results

### LY411,575 reduces the load of *P. berghei* ANKA in hepatic cells in a dose-dependent manner

**Figure 1:**
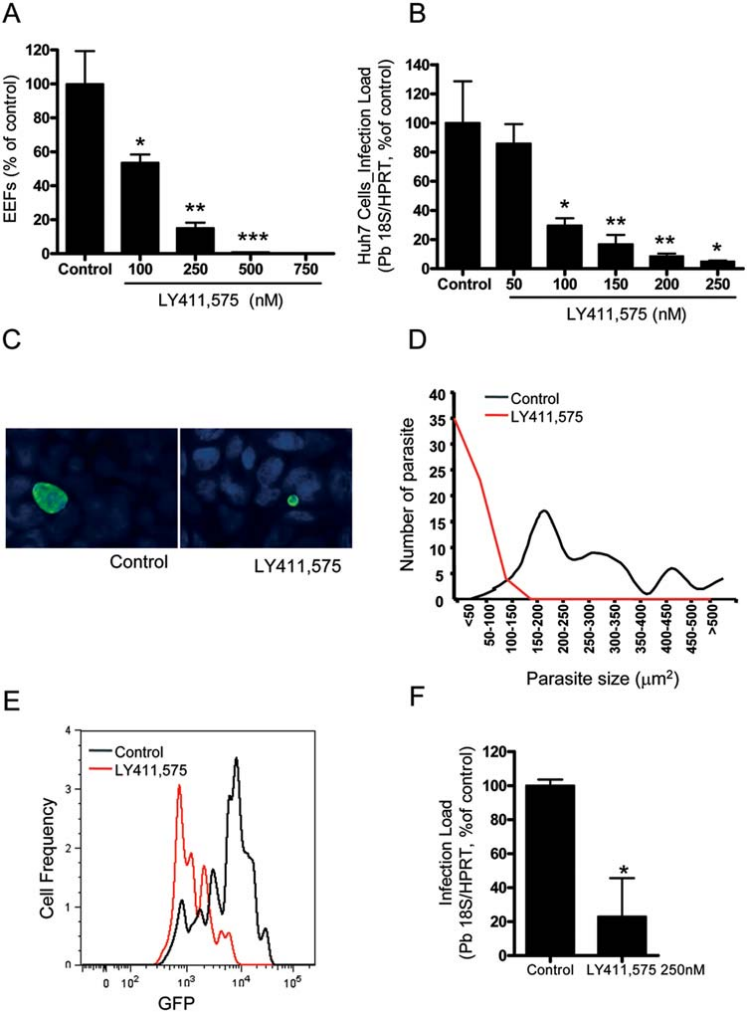
LY411,575 decreases infection of hepatic cells by *P. berghei* ANKA sporozoites. (A, B) Dose-dependent effect of LY411,575 on infection of Huh7 cells, measured by immunofluorescence microscopy (A) or qRT-PCR (B). Control cells were treated with an amount of DMSO equivalent to that of the highest drug concentration and infection was measured 24 h after addition of 20000 *P. berghei* ANKA sporozoites. Experiments were conducted in triplicates. Results are plotted as percentages of the mean value of the control samples (A) (*: p<0.02, **: p<0.01, ***: p<0.001) or as parasite-specific 18S rRNA as measured by qRT PCR (B) (Black circles represent the mean of *P. berghei* ANKA18S rRNA expression in each condition, *n* = 3). (C) Representative images of EEFs in Huh7 cells treated for 48 h with 100 nM LY411,575 and solvent-treated control cells. EEFs were stained for *P. berghei* HSP70 (green) and nuclei were stained with DAPI (blue). (D) Size distribution of EEFs in Huh7 cells treated for 48 h with 100 nM LY411,575 and solvent-treated control cells. Pictures of 50 EEFs were taken from each coverslip and the size of the EEFs was measured using the ImageJ software. (E) Representative lines of GFP intensity of Huh7 cells treated with 250 nM LY411,575 and solvent-treated control cells 30 h after addition of 20000 GFP-expressing *P. berghei* ANKA sporozoites, analyzed by FACS. (F) Effect of LY411,575 on infection of mouse primary hepatocytes, measured by qRT-PCR 48 h after addition of 25000 *P. berghei* ANKA sporozoites. Control cells were treated with an equivalent amount of DMSO. Experiments were conducted in triplicates. Results are plotted as percentages of the mean value of the control samples. *: p<0.02.

In order to measure the influence of LY411,575 on *P. berghei* development monolayers of human hepatoma Huh7 cells cultured in 24-well tissue culture plates, were treated with concentrations of the inhibitor ranging from 100 to 750 nM. Control cells were incubated with medium contaning 0.01% DMSO. Cells were infected with *P. berghei* ANKA sporozoites immediately after addition of the inhibitor. Twenty-four h after infection cells were either fixed with PFA and stained for *P. berghei* HSP70 or lysed in RLT buffer (Qiagen RNeasy Micro Kit) and used for RNA isolation. Infection was quantified by counting the number of infected cells (exo-erythrocytic forms, EEFs) per well, by qRT-PCR or by FACS. The inhibitor blocked the development of the parasites in a dose-dependent manner. Concentrations as low as 100 nM reduced the number of EEF-containing cells, as detected and counted by microscopy, by 45%. At concentrations above 500 nM no EEFs could be detected by microscopy (Fig. 1A). The IC_50_ of LY411,575 calculated on the basis on infection measurement by qRT-PCR (Fig. 1B) was ∼80 nM.

The inhibitor did not only reduce the number of EEFs but also their size (Fig. 1 C,D,E). After 48 h treatment with LY411,575 the average size of the EEFs in cells treated with the drug was 5 to 6 times smaller than in control cells (Fig 1C,D). Furthermore, cells treated with 250 nM LY411,575 and analyzed 30 h after sporozoite addition by FACS showed a greatly reduced average GFP fluorescence intensity per EEF, compared to controls (Fig 1E). Since GFP in these parasites is expressed under the control of the *P. berghei* house keeping gene (EF1α) promoter region, the results are consistent with the presence of smaller and less developed EEFs in the presence of LY411,575. In addition, the infection load in mouse primary hepatocytes treated with the same amount of LY411,575 and analyzed 48 h after sporozoite addition by qRT-PCR was ∼80% lower than that of control cells (Fig 1F), showing that the compound inhibits infection *ex vivo* as well as *in vitro*.

### LY411,575 acts on the early developmental stages of the parasites but does not affect their entry in cells

**Figure 2:**
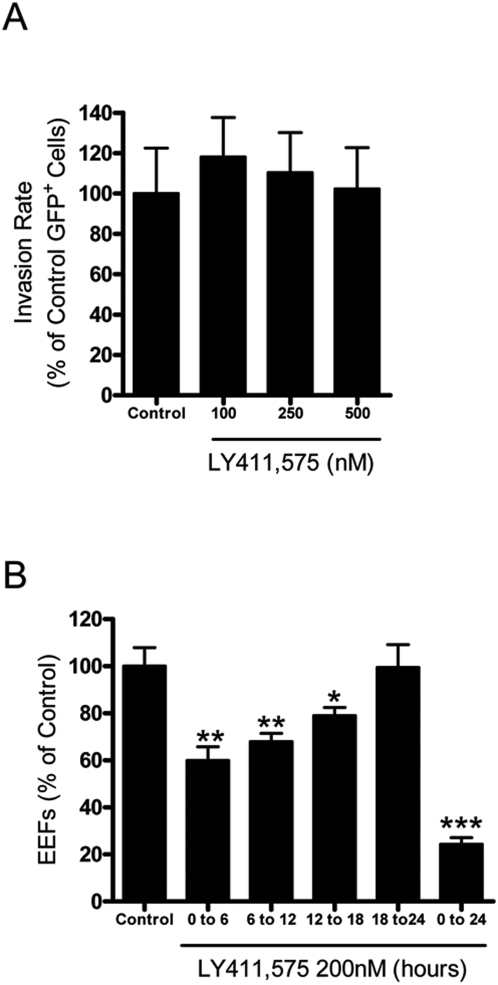
LY411,575 affects development, but not invasion, of *P. berghei* parasites. (A) Effect of the incubation of Huh7 cells with various amounts of LY411,575 on the percentage of GFP-positive cells collected 2 h after addition of 20000 GFP-expressing *P. berghei* ANKA sporozoites and analysed by FACS. Control cells were treated with an amount of DMSO equivalent to that of the highest drug concentration. Experiments were conducted in triplicates. Results are plotted as percentages of the mean value of the control samples. (B) Effect of the incubation of Huh7 cells with 100 nM LY411,575 during various 6 hour intervals and for 24 h. Infection was measured by immunofluorescence microscopy 24 h after addition of 20000 *P. berghei* ANKA sporozoites. Control cells were treated with equivalent amounts of DMSO. Experiments were conducted in triplicates. Results are plotted as percentages of the mean value of the control samples. *: p<0.02, **: p<0.01, ***: p<0.001.

Hepatocyte infection by *Plasmodium* may be conceptually divided into two consecutive steps: hepatocyte invasion and intracellular parasite development. In our *in vitro* infection model, over 95% of the infective sporozoites have completed the migration and invasion steps at 2 h after addition to the cells [15]. The overall effect of LY411,575 on infection observed above could be due to an interference of the drug with invasion of the host cells by the sporozoites, with the subsequent parasite development or with both these processes. The experimental design used above could not allow us to distinguish between these possibilities. To determine whether LY411,575 was affecting sporozoite invasion, we treated cells with 100 to 500 nM of LY411,575 1 h prior to and during the initial 2 h of infection with GFP-expressing *P. berghei* sporozoites. Cells were then collected 2 h after sporozoite addition and the infection level was analyzed by FACS. The percentage of infected cells in the treated samples was similar to that found in control cells, indicating that the compound does not interfere with the invasion process (Fig. 2A). Thus, the LY411,575-mediated decrease on infection depicted in Fig. 1 results solely from an interference of the drug with the intracellular development of the parasite, an effect that is clearly visible when infection was monitored 30 or 48 h after sporozoite addition (see Fig. 1 C,D,E). To test the influence of LY411,575 on the different stages of development of *P. berghei* ANKA Huh7 cells were treated with LY411,575 for periods of 6 h and infection level was measured by microscopy 24 h after sporozoite addition (Fig. 2B). The observed decrease in the number of EEFs in the samples treated during the initial period of infection clearly suggests that LY411,575 affects the *P. berghei* growth during the early developmental stages.

### The effect of LY411,575 on *Plasmodium* development is not due to an interference with the cellular Notch signaling pathway

The effect of LY411,575 on parasite development could be due to inhibition of the function of one or more of its known targets. Well-established targets of this inhibitor are the cellular gamma-secretase complex [16], [17] (and, subsequently, the downstream Notch signaling pathway) and the cellular signal peptide peptidase (SPP) [18]. Importantly, LY411,575 was recently shown to effectively block the activity of the *P. falciparum* SPP homologue in *in vitro* activity assays [19]. Furthermore, our analysis of the *Plasmodium* SPP homologous sequences listed in PlasmoDB showed that all *Plasmodium* species, including *P. berghei*, contain a single SPP homologue.

**Figure 3:**
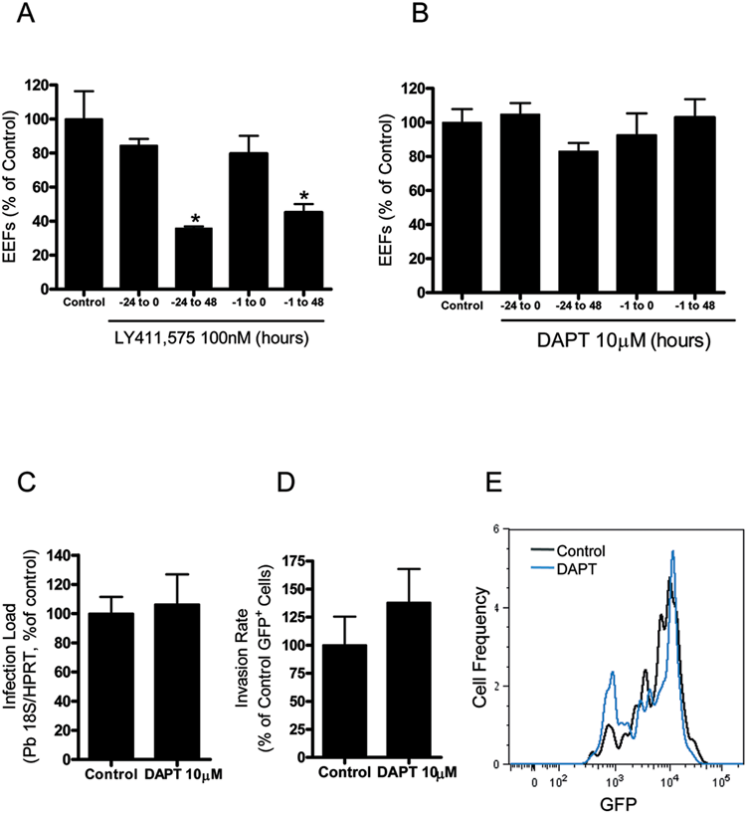
*Plasmodium* growth inhibition is not due to an interference with the cellular Notch signaling pathway. (A) Effect of the incubation of Huh7 cells with 100 nM LY411,575 for different periods relative to sporozoite addition. Infection was measured by immunofluorescence microscopy 48 h after addition of 20000 *P. berghei* ANKA sporozoites. Control cells were treated with an equivalent amount of DMSO. Experiments were conducted in triplicates. Results are plotted as percentages of the mean value of the control samples. *: p<0.05. (B, C) Effect of 10 µM DAPT on infection of Huh7 cells, measured by immunofluorescence microscopy (B) or qRT-PCR (C) 30 h after addition of 20000 *P. berghei* ANKA sporozoites. Control cells were treated with an equivalent amount of DMSO. Experiments were conducted in triplicates. Results are plotted as percentages of the mean value of the control samples. (D) Effect of the incubation of Huh7 cells with 10 µM DAPT on the percentage of GFP-positive cells collected 2 h after addition of 20000 GFP-expressing *P. berghei* ANKA sporozoites and analysed by FACS. Control cells were treated with an amount of DMSO equivalent to that of the highest drug concentration. Experiments were conducted in triplicates. Results are plotted as percentages of the mean value of the control samples. (E) Representative lines of GFP intensity of Huh7 cells treated with 10 µM DAPT and solvent-treated control cells 30 h after addition of 20000 GFP-expressing *P. berghei* ANKA sporozoites and analysed by FACS.

In order to distinguish whether the observed effect of LY411,575 was exerted on the host cell or on the parasite, target cells were treated during the time periods shown on Fig. 3A. As can be seen on this figure, pretreatment of the cells with LY411,575 was not sufficient to block parasite development. The effect of the inhibitor was only detected when the compound was present in the culture medium during the process of development. We therefore concluded that the effect of the inhibitor is most likely on the parasite itself and not due to influence on cellular signaling through gamma-secretase or on cellular SPP.

Additional evidence for the independence of the LY411,575 effect on *Plasmodium* development from the cellular Notch signaling pathway was obtained by experiments with the gamma-secretase inhibitor DAPT. DAPT was previously shown to specifically block gamma-secretase but not SPP [18]. It is well established that 1 µM DAPT is sufficient to completely block the activity of the gamma-secretase in cellular assays [20]. Incubation of Huh7 cells with 10 µM DAPT for various periods did not affect the number of cells containing EEFs detectable by microscopy (Fig. 3B). Ten µM DAPT also had no effect on infection of primary hepatocytes by *P. berghei* ANKA measured by qRT-PCR (Fig. 3C). When cells treated with DAPT were analyzed by FACS at 2 h (Fig. 3D) and 30 h (Fig. 3E) after addition of GFP-expressing sporozoites, no significant differences were observed in the percentage of GFP-positive cells or in GFP intensity, respectively, relative to controls. We therefore concluded that the *Plasmodium* growth inhibition caused by LY411,575 is not due to inhibtion of host gamma-secretase activity.

### LY411,575 reduces development of *P. berghei* ANKA in mouse livers

**Figure 4:**
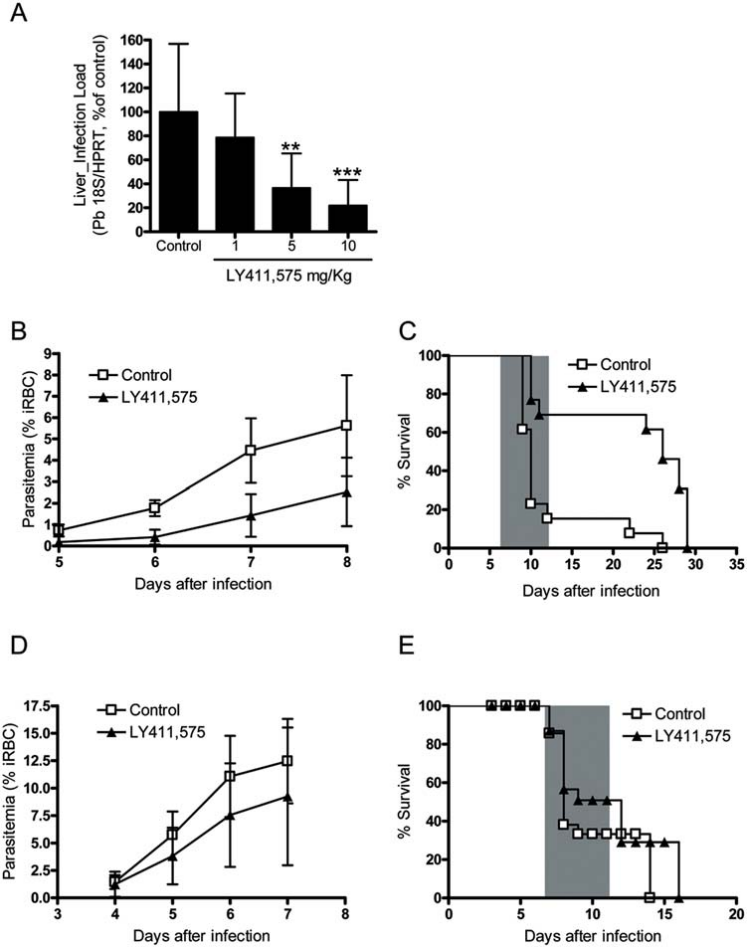
LY411,575 decreases liver *P. berghei* infection *in vivo* and increases CM survival. (A) Dose-dependent effect of LY411,575 on liver parasite load, measured by qRT-PCR. Control mice were treated with an equivalent amount of DMSO and infection was measured on livers collected 40 h after injection of 20000 *P. berghei* ANKA sporozoites (n = 12 for each group). **: p<0.01, ***: p<0.001. (B) Effect of i.p. injection of 10 mg/kg body weight LY411,575 on blood parasitemia of mice infected with 1000 *P. berghei* ANKA sporozoites. Control mice were treated with an equivalent amount of DMSO and mice were monitored daily for parasite levels in the blood and disease symptoms. (n = 13 for each group). Parasitemias on each assessed day are significantly different between the two experimental groups (p<0.001). (C) Survival curves of mice treated by i.p. injection of 10 mg/kg body weight LY411,575 and solvent-treated, control mice, infected with 1000 *P. berghei* ANKA sporozoites. The shaded area represents the time-window for death with CM symptoms. The two survival curves are significantly different (p<0.01). (D) Effect of i.p. injection of 10 mg/kg body weight LY411,575 on blood parasitemia of C57BL/6 mice infected with 50000 iRBC. Control mice were treated with an equivalent amount of DMSO and mice (n = 15 for each group) were monitored daily for parasite levels in the blood and disease symptoms. Parasitemias were not found to be significantly different: p = 0,5375 (day 4); p = 0,0345 (day 5); p = 0,03065 (day 6); p = 0,1446 (day 7). (E) Survival curves of mice treated by i.p. injection of 10 mg/kg body weight LY411,575 and solvent-treated, control mice, infected with 50000 iRBC sporozoites. The shaded area represents the time-window for death with CM symptoms. The two survival curves are not significantly different (p = 0.4474).

In order to analyze the *in vivo* effect of LY411,575, C57BL/6 mice were treated by intraperitoneal (i.p.) injection of the inhibitor in doses ranging from 1 to 10 mg/kg body weight and infected with 20000 *P. berghei* ANKA sporozoites. The mice received two injections of 100 µl, at 2 h before infection and 24 h later. Control animals were treated with an equivalent amount of DMSO. Forty h after infection the animals were sacrificed, their livers were collected and total parasite load in the livers was measured by qRT-PCR (Fig. 4A). The results clearly show that treatment with LY411,575 leads to a dose-dependent reduction in liver parasite load. We therefore hypothesized that the reduced *Plasmodium* liver load in LY411,575-treated mice might have a positive influence on the further development of disease in these animals. To test this hypothesis, C57BL/6 mice were treated with 10 mg/kg body weight of the inhibitor, infected with 1000 *P. berghei* ANKA sporozoites and disease progression was followed. The animals received two injections of LY411,575, as previously described, and were monitored daily for symptoms of CM and death. Blood parasitemia (percentage of infected red blood cells) was measured from day 5 after infection by FACS. LY411,575-treated mice developed significantly lower levels of blood parasitemia when compared to control animals, very evident during the days before development of severe symptoms (Fig. 4B). The drug treatment also influenced significantly the incidence of CM, with LY411,575-treated mice displaying 55% increase in CM survival when compared to control animals (Fig. 4C). To determine whether the observed effect on CM was due to direct influence of LY411,575 on development of *P. berghei* ANKA blood stages or instead just due to the decreased caused in liver infection, C57BL/6 mice were treated with 10 mg/kg body weight of the inhibitor, infected with 5×10^4^ *P. berghei*-infected red blood cells (iRBCs) and disease progression was followed. The animals received three i.p. injections of 50 µl, at 2 h before infection with iRBC and at days 2 and day 4 after infection. Control animals were treated with the equivalent amounts of DMSO. Mice were monitored daily for symptoms of CM and death. Blood parasitemia was measured from day 3 after infection by FACS. Treated mice displayed slightly lower parasitemia levels (Fig. 4D). This small difference was not sufficient to influence the development of CM with both groups of mice showing similar survival curves (Fig. 4E). Altogether these data suggest that LY411,575 alters the course of the blood stage of infection by reducing significantly *Plasmodium* liver stage development.

## Discussion

Despite the attempts for its eradication, malaria is still among the most deadly diseases in the world. During the last 20 to 30 years *Plasmodium* parasites, the causative agent of the disease, have developed resistance to the major groups of antimalarial drugs currently in use. Therefore new antimalarials with novel mechanisms of action are urgently needed.

A few families of *Plasmodium* proteases have emerged during the last couple of years as new potential targets for treatment of malaria, the best characterized among these being the plasmepsins and falcipains. Different classes of inhibitors of these proteases have been developed that show promising results in both *Plasmodium* culture and mouse models [9]–[12]. HIV protease inhibitors currently in clinical use also show antimalarial activity [13], [14].

Here we report that LY411,575 reduces development of *P. berghei* ANKA in human hepatoma cells, primary hepatocytes and mouse livers. Though developed as a potent GSI, LY411,575 also blocks the activity of all human SPP homologues and *P. falciparum* SPP with reasonable potency [18], [19]. LY411575 is not unique and one of many compounds developed as potential therapeutic agents for the treatment of Alzheimer's disease (AD) that appear capable of blocking GSI and SPP activity. Indeed, at least two GSIs are currently being tested as in the clinic [21], [22].

SPP, the prototypic member of the human SPP family of aspartyl proteases catalyzes intramembrane proteolysis of some signal peptides after they have been cleaved from a preprotein and also of several viral preproteins [23]–[25]. SPPL2a and SPPL2b were recently shown to promote intramembrane cleavage of TNF-alpha in activated dendritic cells [26]. Little is known about the functions of SPP homologues in other organisms but there is increasing suggestive evidence that these molecules play critical role in development in a number of species. *Caenorhabditis elegans* deficient for the SPP homologue *ce*-imp2 shows a very severe developmental phenotype [27]. In *Drosophila*, deficiency for CG11840, one of the two SPP homologues present in this organism, leads to death during larval stage due to problems during tracheal development [28]. In *Danio rerio*, knock down of either SPP or SPPL3 homologues results in cell death in the central nervous system; knock down of SPPL2b in the same organism leads to caudal vein enlargement [29].

We hypothesized that the *Plasmodium* SPP homologue could be required for development of the parasite in the host cell and inhibitors of this protease could block the developmental process. Consistent with previous studies [19] analysis of the SPP homologous sequences present in PlasmoDB showed that a single SPP-like protein is present in each of the *Plasmodium* species including the rodent parasite *P. berghei* (acc. numbers PF14_0543, PB001192.00.0 and PY06507 for *P. falciparum*, *P. berghei* and *P. yoelii*, respectively). Moreover, relative expression profiles show that *P. yoelii* and *P. berghei* SPP is equally expressed in both in liver and blood stages (http://plasmodb.org/plasmo/ and our own data not shown, respectively). As selective SPP inhibitors have not been developed for *in vivo* use, we tested our hypothesis using LY411,575, a compound developed as a gamma-secretase inhibitor that also inhibits SPPs including *P. falciparum* SPP [19].

We could clearly show that LY411,575 efficiently blocked the development of *P. berghei* ANKA in hepatic cells in a dose-dependent manner with an IC_50_ of ∼80 nM (Fig. 1). Our detailed analysis of the LY411,575 effect on *Plasmodium* showed that the inhibitor blocked the early stages of parasite development (Fig. 2B). The effect of LY411,575 on *Plasmodium* development could be due to inhibition of SPP activity but also potentially to an interference with cellular host Notch signaling as a result of inhibition of the cellular gamma-secretase complex. We excluded the latter possibility by treating cells with the gamma-secretase specific inhibitor DAPT, which had no effect on *P. berghei* ANKA development (Fig. 3B, C, D, E). Furthermore, pretreatment of cells with LY411,575 did not affect the development of the parasites (Fig. 3A). The drug inhibited development only when present throughout the process. These data seem to suggest that the effect of LY411,575 on *Plasmodium* development is probably due to an effect on the parasite itself and not on the host cell.

We could not detect any effect of LY411,575 on invasion of hepatocytes by *P. berghei* sporozoites. This seems surprising considering the recently published results from Li et al. [30], which show that a SPP antibody generated by them blocks RBC invasion by *P. falciparum in vitro*. However, we consider that our results are difficult to compare to that study because of the differences in the systems used. Nevertheless, we can state that doses of LY411,575 sufficient to partially or completely block *P. berghei* development have no effect on invasion of hepatocytes by the parasite. At the current stage of this study we cannot yet prove that SPP is the only *Plasmodium* target responsible for the observed LY411,575 effect. It is possible that the mechanism underlying the effect of LY411,575 on parasite development is complex and involves inhibition of more than one aspartyl protease. Further experiments will be needed to clarify this issue.

Notably, LY411,575 also showed promising results against *Plasmodium* liver stage development in mice treated with this compound. LY411,575-treated C57BL/6 mice displayed a decrease in *Plasmodium* liver load, which later manifested in lower blood parasitemia levels. In addition, the drug treatment significantly influenced the development of CM, with a 55% higher mortality in the control group of animals compared to the LY411,575-treated ones. The LY411,575 treatment scheme used in this study provided only partial protection of mice against *P. berghei* infection. Nevertheless, because the effect of the drug is clearly dose-dependent in both cells and mice, we expect that higher doses or longer treatment would lead to even greater reduction in liver load and parasitemia and possibly to destruction of all parasites by the drug in the liver. Notably, even such partial decrease of liver load and blood parasitemia led to a significant reduction of incidence of development of severe symptoms (CM) in the treated group of animals. The effect of LY411,575 on the development of *P. berghei* blood stages was much less pronounced. However, the relatively small influence of LY411,575 on *P. berghei* blood stages does not diminish the importance of our results. Indeed, this may only reflect the availability of the drug in the liver *versus* in circulation after i.p. administration of this specific dosage. Nevertheless, we can conclude that LY411,575 is a potent inhibitor of development of *P. berghei* in the liver.

For nearly a decade there has been a substantive effort to develop GSIs which would reduce the production of the amyloid β peptide that accumulates in the AD patient's brain [31], [32]. Although toxicity (largely due to inhibition of Notch signaling) has constituted an obstacle to the clinical development of GSIs for AD, one compound remains in a phase III human trial for AD, and other compounds are being tested as anti-cancer agents. In the latter case, the rationale is that several cancers have been shown to be dependent on Notch signaling. Thus, a large array of LY411,575-related and other non structurally related GSIs already exists, many of which also inhibit SPP. A very promising approach to the development novel anti-malarial agents would be to leverage the GSI drug-discovery to find compounds that are drug-like and inhibit SPP selectively or even more preferentially *Plasmodium* SPP. Indeed, the fact that LY411,575 inhibits both SPP and GS limits the current study in terms of efficacy. Higher doses, which might be more effective against blood-stages, are not well tolerated due to GS toxicity related to the GSI activity.

## Materials and Methods

### Mice, cells and parasites

Male C57BL/6 mice were bred and housed in the specific pathogen free facilities of the Instituto Gulbenkian de Ciência (IGC) according to the guidelines of the Animal Care Committee of the IGC. All mice used were 6 to 8 weeks old.

Human hepatoma Huh7 cells (ATCC CCL-185) were cultured in RPMI medium supplemented with 10% FCS, 1 mM glutamine, 1% non-essential aminoacids, 1% penicillin/streptomycin, 10 mM Hepes buffer.

Green fluorescent protein (GFP)-expressing *P. berghei* ANKA (parasite line 259cl2, which shows similar virulence to wild-type *P. berghei*) [33] sporozoites were obtained from dissection of infected *Anopheles stephensi* mosquito salivary glands at day 21–25 post-infection, which were produced and maintained at IMM insectary.

### Isolation of murine primary hepatocytes

Mouse primary hepatocytes were isolated from C57BL/6 mice as previously described [34]. Briefly, cells were obtained by perfusion of mouse liver lobules with liver perfusion and liver digest medium (Gibco) at 37°C. Hepatocytes were then purified using 1.12 g/ml, 1.08 g/ml and 1.06 g/ml Percoll gradients. Cells were cultured in William's E medium (Gibco) containing 4% FCS, 1% penicillin/streptomycin in 24-well plates coated with 0.2% Gelatin in PBS.

### LY411,575 and DAPT treatment and infection *in vitro*

LY411,575 was synthesized as previously described [35]. The compound was reconstituted in DMSO to obtain a 10 mM stock solution. DAPT (N-[N-(3,5-Difluorophenacetyl-L-alanyl)]-S-phenylglycine t-Butyl Ester; gamma-secretase inhibitor IX) was purchased from Calbiochem (Darmstadt, Germany) as a 25 mM solution in DMSO.

Huh7 human hepatoma cells were treated with LY411,575 or DAPT diluted in culture medium as described in the text. Control cells were incubated with medium containing 0.01% or 0.04% DMSO, respectively. Twenty thousand *P. berghei* ANKA sporozoites were added to monolayers of 1.5×10^5^ Huh7 cells cultured on glass coverslips or directly in 24-well tissue culture plates and centrifuged for 5 min at 3000 rpm. Cells used for immunofluorescence staining were fixed 24 or 48 h after infection and stained for *P. berghei* HSP70. Infection was quantified by counting the number of infected cells (exoerythrocytic forms, EEFs) per coverslip. Huh 7 cells and primary hepatocytes used for qRT-PCR were lysed in RLT buffer (Qiagen RNeasy Micro Kit) at 24 and 48 h after infection, respectively, and processed according to manufacturer's guidelines. Huh7 cells used for fluorescence activated cell sorting (FACS) analysis were collected at 2 and 30 h after sporozoite infection.

### LY411,575 treatment and infection *in vivo*

C57BL/6 mice were injected intraperitoneally (i.p.) with 1 to 10 mg/kg body weight (average body weight 20 mg) LY411,575 in 100 µl DMSO. Control animals were treated with the same amount of DMSO. All mice received two injections, the first one 2 h before infection with *P. berghei* ANKA sporozoites and the second one 24 h later. Mice were infected by intravenous (i.v.) injection of 20000 (for parasite liver load measurement) or 1000 (for survival assessment) *P. berghei* ANKA sporozoites. Parasite load in the liver was measured 40 h after infection by qRT PCR.

Mice monitored for survival after infected blood (iRBC) challenge were injected i.p. with 10 mg/kg body weight LY411,575 in 50 µl DMSO. Control animals were treated with the same amount of DMSO. All mice received three injections, the first one 2 h before infection with iRBC and the second and third ones at day 2 and day 4 after infection, respectively. Mice were infected by intraperitoneal injection of 50000 iRBC.

During survival experiments, mice were monitored every day for disease symptoms and time of death. Parasitemias (percentage of infected red blood cells) were measured daily by Flow Cytometry on FACSCalibur.

### Immunofluorescence

Huh7 cells were fixed with 4% paraformaldehyde (PFA) in PBS for 20 min and incubated in blocking buffer (3% BSA, 10% FCS, 100 mM glycine, 0.1% saponin in PBS) for 1 h followed by incubation with monoclonal antibody 2E6 against *P. berghei* HSP70 [36] diluted in the same buffer. Cells were then washed with 0.1% saponin in PBS and incubated with a secondary antibody diluted in blocking buffer (Anti-Mouse Alexa488, Molecular Probes) for 30 min. Nuclei were stained with DAPI. Images were acquired with a Leica DM5000B fluorescence microscope and processed using Adobe Photoshop.

### Infection quantification by qRT-PCR

For infection determination *in vivo* or *ex vivo*, total RNA was isolated from livers or primary hepatocytes using Qiagen's RNeasy Mini or Micro kits, respectively, following the manufacturer's instructions. The assessment of liver parasite load *in vivo*, was performed according to the method developed for *P. yoelii* infections [37]. Livers were collected and homogenized in denaturing solution (4 M guanidine thiocyanate, 25 mM sodium citrate pH 7, 0.5% sarcosyl and 0.7% β-Mercaptoethanol in DEPC-treated water) 40 h after sporozoite injection. Total RNA was extracted using Qiagen's RNeasy Mini kit. RNA for infection measurements was converted into cDNA using Transcriptor First Strand cDNA Synthesis kit from Roche. The qRT-PCR reactions used Applied Biosystems' Power SYBR Green PCR Master Mix and were performed on an ABI Prism 7000 system (Applied Biosystems). Amplification reactions were carried out in a total reaction volume of 25 µl, containing 0.8 pmol/µl or 0.16 pmol/µl of PbA 18 S- or mouse Hypoxanthine Guanine Phosphoribosyltransferase (HPRT) specific primers, respectively. Relative amount of PbA mRNA was normalized against the HPRT level in each sample. PbA 18 S- and mouse HPRT-specific primer sequences were 5′- AAG CAT TAA ATA AAG CGA ATA CAT CCT TAC - 3′ and 5′ - GGA GAT TGG TTT TGA CGT TTA TGT G - 3′and 5′ - TGC TCG AGA TGT GAT GAA GG - 3′ and 5′ - TCC CCT GTT GAC TGG TCA TT - 3′, respectively.

### Fluorescence Activated Cell Sorting (FACS) analysis

FACS analysis was performed to determine the percentage of parasite-containing cells at 2 and 30 h after sporozoite addition as described in [15]. Briefly, cells were washed with PBS, detached by trypsin treatment and collected in 400 ul 10% FCS in PBS at the selected time points after sporozoite addition. Cells were then centrifuged at 0.1 rcf for 3 min at 4°C, resuspended in 150 ul 2% FCS in PBS and analyzed on a Becton Dickinson FACScalibur. Data acquisition and analysis were carried out using CELLQuest (version 3.2.1fl1, Becton Dickinson) and FlowJo (version 6.3.4, FlowJo) software.
